# Avian Reovirus P17 Suppresses Angiogenesis by Promoting DPP4 Secretion

**DOI:** 10.3390/cells10020259

**Published:** 2021-01-28

**Authors:** Ekta Manocha, Antonella Bugatti, Mirella Belleri, Alberto Zani, Stefania Marsico, Francesca Caccuri, Marco Presta, Arnaldo Caruso

**Affiliations:** 1 Section of Microbiology, Department of Molecular and Translational Medicine, University of Brescia, 25123 Brescia, Italy; e.manocha@unibs.it (E.M.); antonella.bugatti@unibs.it (A.B.); a.zani033@unibs.it (A.Z.); francesca.caccuri@unibs.it (F.C.); 2 Section of Experimental Oncology and Immunology, Department of Molecular and Translational Medicine, University of Brescia, 25123 Brescia, Italy; mirella.belleri@unibs.it (M.B.); marco.presta@unibs.it (M.P.); 3 Department of Pharmacy, Health and Nutritional Sciences, University of Calabria, Arcavacata di Rende, 87036 Cosenza, Italy; stefania.marsico@unical.it

**Keywords:** ARV p17, angiogenesis, DPP4, VEGF-A, FGF-2, antiangiogenic activity

## Abstract

Avian reovirus p17 (ARV p17) is a non-structural protein known to activate autophagy, interfere with gene transcription and induce a significant tumor cell growth inhibition in vitro and in vivo. In this study, we show that ARV p17 is capable of exerting potent antiangiogenic properties. The viral protein significantly inhibited the physiological angiogenesis of human endothelial cells (ECs) by affecting migration, capillary-like structure and new vessel formation. ARV p17 was not only able to suppress the EC physiological angiogenesis but also rendered ECs insensitive to two different potent proangiogenic inducers, such as VEGF-A and FGF-2 in the three-dimensional (3D) Matrigel and spheroid assay. ARV p17 was found to exert its antiangiogenic activity by upregulating transcription and release of the well-known tumor suppressor molecule dipeptidyl peptidase 4 (DPP4). The ability of ARV p17 to impact on angiogenesis is completely new and highlights the “two compartments” activity of the viral protein that is expected to hamper the tumor parenchymal/stromal crosstalk. The complex antitumor activities of ARV p17 open the way to a new promising field of research aimed to develop new therapeutic approaches for treating tumor and cancer metastasis.

## 1. Introduction

Avian reoviruses (ARVs) are important pathogens, belonging to the Orthoreovirus genus in the Reoviridae family, associated with different diseases, like viral arthritis, enteric disease problems, immunosuppression and chronic respiratory disorders in many avian species [1]. The ARV genome consists of 10 double-stranded RNA-genome segments, which encode at least 10 structural proteins and four nonstructural proteins [2], but very little is known about the functions of most of the proteins. The non-structural p17 protein of ARVs (ARV p17) is a 146 amino acid (aa) protein encoded by the S1 gene segment, whose Open Reading Frame (ORF) is highly conserved in all avian Reovirus S1 segments and has been suggested to play an important role in virus–host interaction [3]. The ARV p17 does not show sequence similarity with any other known protein, so its sequence offers no clues about its function [4]. It is known to be a nucleocytoplasmic shuttling protein, which has been suggested to participate in cell nuclear processes, such as gene transcription, DNA binding and cell growth regulation [5,6]. The viral protein possesses the capability to translocate into the nucleus, induce autophagy and increase viral replication [7,8]. Recently, different studies have reported the capability of ARV p17 to promote a significant cell growth inhibition and cell cycle retardation in several cancer cell lines through activation of the p53 pathway [5,9] and interaction with cyclin-dependent kinases (CDKs) and cyclins [6,10]. In particular, the direct interaction of ARV p17 with CDK1 leads to its inactivation, but also to the suppression of the Serine/Threonine-protein kinase Plk1, an early trigger for G2/M transition with oncogenic properties [11,12].

Tumor growth, progression and metastasis are driven by angiogenesis [13], and inhibition of angiogenesis has become one of the most exciting approaches in the development of anticancer therapy. Several viral structural and non-structural proteins are able to induce cell cycle arrest, display anticancer activity in vitro and in vivo [14,15,16,17,18] and concomitantly, potent antiangiogenic functions [19]. In this study, we investigate the properties of ARV p17 in regulating angiogenesis and demonstrate that the viral protein is able to significantly inhibit macrovascular and microvascular EC migration, capillary-like structure and sprouting angiogenesis in a three-dimensional (3D) EC organotypic culture. Moreover, we highlight that the antiangiogenic activity of the viral protein is exerted through the increased secretion of dipeptidyl peptidase (DPP4), a molecule known to impair the pivotal role of the CXCL12/CXCR4 axis in angiogenesis [20,21,22].

## 2. Materials and Methods

### 2.1. Cell Cultures

Human umbilical vein endothelial cells (HUVECs) were isolated and characterized as previously described [23]. Cells were cultured in endothelial cell growth medium (EGM MV; Promo cell, Heidelberg, Germany) supplemented with 10% (vol/vol) Fetal Bovine Serum (FBS) at 37 °C in a humidified atmosphere of 5% CO_2_. Human lung microvascular endothelial cells (HMVEC-Ls) were purchased from Clonetics (San Diego, CA, USA) and cultured in EGM-2MV (Lonza, Basel, Switzerland) containing 10% FBS and growth factors (EGM-MV2 BulletKit; Lonza). Adherent cells were cultured until 80–90% confluence. All experiments were carried out with cells at passage 2–6.

### 2.2. Cloning, Production and Purification of Recombinant GST-ARV p17

The coding sequence of ARV p17 (accession number: AAK18187.1) has been synthesized (Integrated DNA Technology, Coralville, IA, USA) and cloned into the XBA1 and APA1 sites of the expression vector pVAX1 plasmid (Thermo Fisher Scientific, Waltham, MA, USA).

For recombinant GST-ARV p17 protein purification, the full-length ARV p17 gene was amplified from pVAX1 vector using the following primers: 5′-CGCTCGAGGGATCCATGCAATGGC-3′ (forward) and 5′- GCGGGTTTAAACCTCGAGTCATAGATC-3′ (reverse) (0.2 µM; Integrated DNA Technology), cloned into the prokaryotic expression vector pGEX-4T-1 (GE Healthcare, Chicago, IL, USA) and expressed in the BL21 strain of *Escherichia coli*. Selected bacterial clones were induced with 2 mM IPTG at 30 °C for 3 h and GST-ARV p17 protein was recovered from the insoluble fraction of bacterial lysates, purified under denaturing conditions using 6 M urea (Sigma-Aldrich, St. Louis, MO, USA), followed by an overnight dialysis with a urea gradient in dialysis buffer (EDTA 100 mM, Tris HCl 1 M, DTT 1 M, PMSF 100 mM, 1% Triton X-100). Finally, the recombinant protein was eluted from glutathione-sepharose resin (GE Healthcare) by affinity purification in elution buffer (50 mM Tris, 10 mM reduced glutathione, 5 mM DTT, pH 8.0). Recombinant human GST protein was obtained from Abcam (Cambridge, UK) and was used as a negative control. Visualization of protein bands was done by staining with 0.25% Coomassie brilliant blue R-250 (Bio Rad, Hercules, CA, USA) following separation on a 14% SDS-PAGE gel. The identity of the purified GST-ARV p17 protein was confirmed by Western blotting as follows: Recombinant GST- and GST-ARV p17 protein were separated on a freshly prepared gel and transferred onto polyvinylidene fluoride membrane (PVDF, GE Healthcare). After blocking with 3% BSA (bovine serum albumin) in Tris buffer saline containing 0.01% Tween 20 (TBS-T), the blot was probed with goat anti-GST antibody (1:2000) (GE Healthcare). The antigen–antibody complex was detected using peroxidase-conjugated donkey anti-goat IgG (Thermo Fisher Scientific) and developed using the enhanced chemiluminescence (ECL) system (Santa Cruz Biotechnology, Dallas, TX, USA).

### 2.3. Nucleofection

Nucleoporation of ECs was performed using the Amaxa Nucleofector Technology (Lonza) following the manufacturer’s protocol. Endotoxin-free plasmid expressing ARV p17 (4 µg) was added to 1 × 10^6^ cells resuspended in 100 µL of nucleofection buffer. Mock nucleofected cells (nucleofected with nucleofection solution only) were used as a negative control. Experiments were carried out at 48 h post-nucleofection.

### 2.4. Cell Proliferation Assay

Mock- and ARV p17-nucleofected cells were seeded in 6-well plates at a density of 0.5 × 10^6^ cells/well and passaged 1:2 when they were grown to approximately 80% confluence. At the indicated time points, cells were trypsinized and counted using trypan blue exclusion.

### 2.5. RNA Extraction, PCR and Quantitative Real-Time PCR Analysis

Total RNA was extracted from ECs using the RNeasy Plus Mini Kit (Qiagen, Hilden, Germany) and reverse transcribed (Applied Biosystems, Foster City, CA, USA). The following primers were used to perform PCR (0.2 μM; Integrated DNA Technology) ARV p17: 5′-GCCGGTTCGCTCTCTATTCA-3′ (forward), 5′-ATGGATTGAGACCCGCCATC-3′ (reverse) and β-actin: 5′-GGCACCCAGCACAATGAAG-3′ (forward) and 5′-GCTGATCCACATCTGCTGG-3′ (reverse). The PCR products of 146 and 115 bp respectively, were analyzed on a 2% agarose gel. Quantitative real-time PCR was performed on an ABI Prism 7500 sequence detection system with DPP4 and β-actin TaqMan gene expression assays (Life Technologies, Carlsbad, CA, USA).

### 2.6. Wound Healing Assay

The wound healing assay was performed following previously described procedures with minor modifications [14]. Cells (1 × 10^5^) were plated into collagen-coated 24-well plates overnight. Twenty-four hours later, the monolayer was scratched using a 200 μL pipette tip and cultured in complete medium. The percentage of wound healing was evaluated during a period of 8–10 h. ECs migration was recorded using a DM-IRB microscope system (Leica, Wetzlar, Germany), equipped with a Charge Coupled Device (CCD) camera (Hitachi Ltd., Tokyo, Japan) and connected to a computer via a frame grabber (Matrox Meteor). Analysis of the images was performed using the QWin-lite software (Leica). In some experiments, confluent EC monolayers were pretreated, before scratching, with conditioned medium from Mock- or ARV p17-nucleofected cells for 16 h. In other experiments, EC monolayers were scratched and then cultured in the presence of 10 μM Diprotin A (DPA; Abcam, Cambridge, UK).

### 2.7. Cell Motility Assay

The cell motility assay was performed as previously described [19]. Tissue-culture flasks were coated with collagen (calf skin, Sigma-Aldrich). HUVECs were added to the bottom of the coated flasks at a concentration of 10^5^ cells/flask and allowed to adhere by overnight incubation in an upright position. Flasks were subsequently positioned at a ≈20° angle, so that both the cell-coated surface and the empty surface were immersed in culture medium. Cell motility rates were analyzed at day 10 by measuring the distance from the edge of the flask to the leading edge of the cells and photographed with a Hitachi KP-D50 camera.

### 2.8. Tube Formation Assay

Tube formation assays were performed as previously described [24]. Shortly, 150 μl of Cultrex Basement Membrane Extract (BME; 10 mg/mL) (Trevigen Inc., Gaithersburg, MD, USA) was transferred to prechilled 48-well culture plates. Plates were then incubated for 1 h at 37 °C. Cells were resuspended in EGM or EGM-2 medium (for HUVECs or HMVECs, respectively) containing 10% FBS, seeded 5 × 10^4^ per well, and tube formation was observed over a period of 6 h after cell seeding. The capillary-like structures were photographed with a Hitachi KP-D50 camera and then quantified as number of tubes/well.

In some experiments, a co-culture between Mock- or ARV p17-nucleofected HUVECs was performed using 6-well plates with 0.4 µm pore-size transwell inserts (polycarbonate filters coated with collagen, Corning, New York, USA). Nucleofected cells (1 × 10^6^ cells) were seeded in 2.6 mL EGM with 10% FBS in the lower compartment, while non-nucleofected cells (1.5 × 10^5^ in 1.5 mL EGM with 10% FBS) in the upper chamber. After 48 h of co-cultivation at 37 °C, cells in the upper well were trypsinized and used to perform the tube formation assay. In other experiments, cells were seeded and cultured for tubes formation in the presence of 10 μM DPA.

### 2.9. Matrigel Assay

HUVECs (2 × 10^6^ cells/mL) were mixed with an equal volume of BME (Trevigen Inc.) with Fibroblast Growth Factor 2 (FGF-2) (100 ng/mL) (Santa Cruz Biotechnology) or Vascular Endothelial Growth Factor A (VEGF-A) (100 ng/mL) (Miltenyi Biotec, Bergisch Gladbach, Germany). Each mixture was equally poured in 48-well plates (Corning), in the form of a spot in triplicates per experimental condition. After polymerization of the gel for 1 h at 37 °C, each spot of cells embedded in Matrigel was bathed in 500 µL of complete medium. After 24 h incubation, the tube formation was observed, and the number of closed areas was counted. Images were captured with a Hitachi KP-D50 camera.

### 2.10. Spheroids Assay

Spheroids were generated by mixing ECs (1.5 × 10^5^ cells/mL) with 5 mg/mL of methylcellulose (Sigma-Aldrich) in EGM or EGM-2 medium (for HUVECs or HMVECs, respectively) containing 10% FBS, making the final volume 10 mL. The cells (100 µL/well) were then added to 96-well plates (Greiner Bio-one, Kremsmünster, Austria) and incubated at 37 °C, 5% CO_2_ for 24 h.

Separately, the collagen I gel solution (Rat tail, Corning) was maintained on ice and neutralized by adding NaOH 0.1 M and Phosphate Buffered Saline (PBS) 10× to a final pH of 7.4. Then, the 24-well plates were coated with neutralized collagen (200 µL/well) and incubated in a humidified 5% CO_2_ incubator for 1 h at 37 °C. The spheroids from 96-well plates were collected in Eppendorf tubes and centrifuged at 4000× rpm for 5–10 s. When a clear pellet was distinguished, the supernatant was removed, and the pellet was kept in a volume of about 100 µL collagen I-neutralized solution. Each collagen-spheroid mixture was rapidly added to the precoated 24-well plates at 100 µL/well and incubated for 1 h. After 1 h, 500 µL of conditions (FGF-2 at 100 ng/mL, VEGF-A at 30 ng/mL or DPA at 10 µM) were added to the wells to cover the surface completely and plates were further incubated for 24 h. Sprouting occurred from the spheroid core, photographed with a Hitachi KP-D50 camera, and the sprout number (mean ± standard deviation (SD)) was counted with the spheroids of similar sizes from three different wells of the plate.

### 2.11. Immunofluorescence

Cells treated with GST- or GST-ARV p17 were fixed, permeabilized, blocked with 3% BSA and incubated overnight with a rabbit polyclonal antibody to ARV p17 (dilution of 1:100; Abcam). Then, the cells were washed and incubated with AlexaFluor 488 conjugated goat anti-rabbit IgG secondary antibody (dilution of 1:500; Thermo Fisher Scientific) for 1 h at room temperature. After three washes with PBS, slides were mounted and samples were observed using a Leica (Wetzlar, Germany) TCS SP5 laser scanning fluorescence microscope and the imaging software Leica Application Suite.

### 2.12. Aortic Ring Assay

The assay was performed as previously described [25]. Aortic rings obtained by cross-sectioning the thoracic aorta of 2-month-old C57BL/6 female mice were incubated in serum-free medium in the presence of recombinant GST- or GST-ARV p17 (10 ng/mL). After 24 h, rings were embedded in fibrin gel and incubated with serum-free endothelial cell basal medium (EBM, Clonetics) plus 10 µg/mL aprotinin (Sigma-Aldrich) in the absence or presence of FGF-2 (100 ng/mL). Medium and stimuli were replaced every day. After 6 days, vessel sprouts, morphologically distinguishable from scattering fibroblasts/smooth muscle cells, were counted under a stereomicroscope (STEMI-SR, Zeiss).

### 2.13. Chick Chorioallantoic Membrane (CAM) Assay

Alginate beads (4.0 µL) containing vehicle or 100 ng of FGF-2 with or without GST- or GST-ARV p17 (both at 20 ng/pellet) were prepared as previously described [26] and placed on the top of the embryo CAM of fertilized White Leghorn chicken eggs at day 11 of incubation. After 72 h, newly formed blood micro-vessels converging towards the implant were counted in ovo at 5× magnification under a stereomicroscope (STEMI-SR, Zeiss, Oberkochen, Germany).

### 2.14. Angiogenesis Microarray Analysis

HUVECs were nucleofected and cultured for 24 h, then conditioned medium was collected, clarified and analyzed for the expression of 55 different angiogenesis-related proteins by the Human Angiogenesis Array Kit (Proteome Profiler, R&D systems, Minneapolis, MN, USA) according to the manufacturer’s instructions.

### 2.15. Human DPP4/CD26 Enzyme-Linked Immunosorbent Assay (ELISA)

HUVECs were stimulated with GST- or GST-ARV p17 for 48 h, then conditioned medium was collected, clarified and concentrated with Centricon Amicon YM-30 (cutoff 30 kDa). The release of DPP4 in the concentrated conditioned medium was evaluated using a human CD26 ELISA Kit (R&D systems) according to manufacturer’s instructions. Each sample was analyzed in triplicate.

### 2.16. Statistical Analysis

Data obtained from multiple independent experiments are expressed as mean ± standard deviation (SD). The data were analyzed for statistical significance using Student’s t-test or one-way analysis of variance (ANOVA). Bonferroni’s post-test was used to compare data. Differences were considered significant at *p <* 0.05. Statistical tests were performed using Prism 8 software (GraphPad, San Diego, CA, USA).

## 3. Results

### 3.1. ARV p17 Transduction in HUVECs

**Figure 1:**
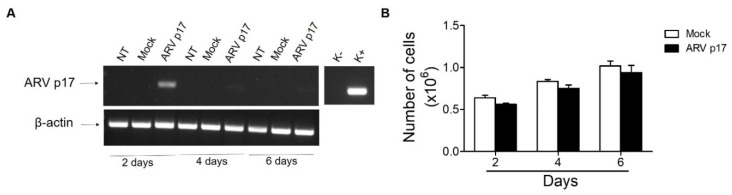
Avian Reovirus p17 (ARV p17) expression in HUVECs. HUVEC cells were mock-nucleofected (Mock) or nucleofected with ARV p17-expressing plasmid (ARV p17). (**A**) The presence of ARV p17 mRNA was analyzed by RT-PCR at different days post-nucleofection. K^-^, negative control, water; K^+^, positive control, ARV p17-expressing plasmid. As a control, amplification of β-actin mRNA is also shown. (**B**) At each time point, cells were counted using the trypan blue exclusion method. Values represent the mean ± standard deviation (SD) of one representative experiment out of three with similar results, performed in triplicates.

HUVECs were nucleofected with the nucleofection solution alone (Mock) or with a plasmid vector pVAX harboring the ARV p17 DNA (ARV p17). As shown in Figure 1A, ARV p17 expression is transient in transduced cells, with maximal expression being observed at day 2 after nucleofection, to decrease thereafter. Notably, ARV p17 transduction did not affect the proliferation of HUVECs. In fact, as shown in Figure 1B, ARV p17-expressing cells did not show any statistically significant difference in doubling time as compared to Mock HUVECs. Based on this data, all the following assays were performed at day 2 post nucleofection.

### 3.2. ARV p17 Expression Inhibits HUVEC Migration and Morphogenesis

**Figure 2:**
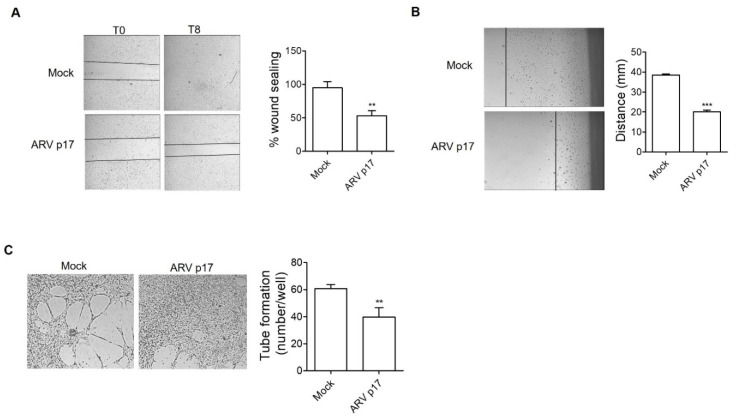
ARV p17 inhibits HUVEC cell migration and morphogenesis. HUVEC cells were mock-nucleofected (Mock) or nucleofected with ARV p17-expressing plasmid (ARV p17). (**A**) ECs’ wound repair ability was analyzed 48 h post-nucleofection. Confluent cell monolayers were scratched using a 200 μL pipette tip and cell migration was recorded by light microscopy for 8 h after wound scratch (original magnification, 4×). The wound width was measured, and the relative wound area was calculated as the ratio of the remaining area at the 8 h time point to the 0 h starting point. Images are representative of one out of three independent experiments with similar results. Values are the mean ± SD of one representative experiment out of three with similar results, performed in triplicates. Statistical analysis was performed by Student’s *t* test. ** *p <* 0.01. (**B**) Cell movement along the bottom of an angled flask was recorded at day 10 post-nucleofection (original magnification, 10×). Cell motility rate was analyzed by measuring the distance from the edge of the flask to the leading edge of the cells. Images are representative of one out of three independent experiments with similar results. Values are the mean ± SD of one representative experiment out of three with similar results, performed in triplicates. Statistical analysis was performed by Student’s *t* test. *** *p <* 0.001. (**C**) HUVECs were seeded on BME-coated plates. Images were taken after 6 h of HUVEC culture on BME (original magnification, 4×). Closed rings were counted as a parameter for quantification of tube formation. Images are representative of one out of three independent experiments with similar results. Values are the mean ± SD of one representative experiment out of three with similar results, performed in triplicates. Statistical analysis was performed by Student’s *t* test. ** *p <* 0.01.

The capability of ARV p17 to interfere with the migratory activity of ECs was assessed by a wound healing assay. Confluent HUVEC monolayers were scratched with a 200 µL tip and the percentage of wound sealing was observed over a period of 8 h. Mock cells reached ~100% of sealing 8 h after the wound, while ARV p17-expressing cells showed a significant inhibition in the capacity to repair the mechanical wound (Figure 2A). Then, to evaluate the effect of ARV p17 expression on long-term migration of ECs, we performed an angled flask migration assay in which the cell movement along the bottom of an angled flask was measured at day 10 post-nucleofection. In keeping with the results obtained in the wound healing assay, cell migration was strongly hampered in ARV p17-expressing cells as compared to Mock cells (Figure 2B). Finally, the ability of ARV p17 to modulate the morphogenic activity of ECs was investigated in vitro by a tube formation assay. To this aim, HUVECs were seeded on 48-well plates (5 × 10^4^/well) containing polymerized plugs of basement membrane extract (Cultrex). As shown in Figure 2C, Mock HUVECs formed a consistent network of capillary-like structures that were almost absent for ARV p17-expressing cells. Taken together, these data highlight the ability of ARV p17 to inhibit HUVEC migration and morphogenesis.

### 3.3. ARV p17 Expression Inhibits FGF-2-Induced Angiogenic Responses in HUVECs

**Figure 3:**
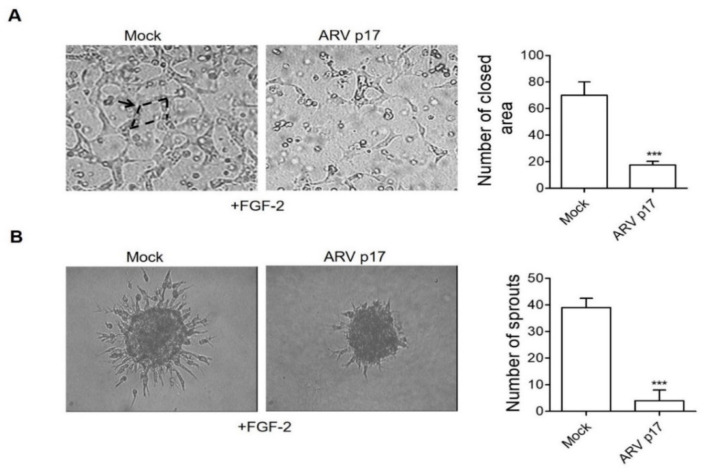
ARV p17 inhibits ECs angiogenesis. HUVEC cells were mock-nucleofected (Mock) or nucleofected with ARV p17-expressing plasmid (ARV p17). Angiogenesis was analyzed 48 h post-nucleofection. (**A**) Tube formation of Mock or ARV p17 HUVECs along with FGF-2 treatment in a three-dimensional (3D) Matrigel culture. Lined by the tubes, the closed areas (an example indicated by the arrow) were counted as a parameter for quantification of tube formation. Pictures were taken after 24 h of culture (original magnification, 10×). Pictures are representative of one out of three independent experiments with similar results. Values are the mean ± SD of one representative experiment out of three with similar results, performed in triplicates. Statistical analysis was performed by Student’s *t* test. *** *p <* 0.001. (**B**) Sprouting of spheroids generated by Mock or ARV p17 HUVECs upon FGF-2 treatment. Pictures were taken after 24 h of culture (original magnification, 20×). The bar graph shows the average number/spheroid of EC sprouts. Pictures are representative of one out of three independent experiments with similar results. Values are the mean ± SD of one representative experiment out of three with similar results, performed in triplicates. Statistical analysis was performed by Student’s *t* test. *** *p <* 0.001.

To assess the capacity of transduced ARV p17 to affect the ability of HUVECs to respond to the stimulation exerted by the prototypic angiogenic mediator Fibroblast Growth factor-2 (FGF-2), Mock and ARV p17 HUVECs were mixed with Matrigel and FGF-2 (100 ng/mL). Within 24 h of incubation, Mock HUVECs migrate and align to form tubes organized in a capillary-like network in this three-dimensional (3D) Matrigel culture. This capacity was significantly impaired in ARV p17-expressing cells (Figure 3A).

EC spheroids embedded in biopolymeric gels can be induced to form endothelial sprouts following stimulation with angiogenic factors, thus representing a 3D cell model that mimics in vivo sprouting angiogenesis [27]. On this basis, Mock and ARV p17 HUVEC-derived spheroids were embedded in a type I collagen gel in the presence of FGF-2. As anticipated, a 24 h stimulation of Mock HUVEC spheroids with FGF-2 strongly promoted micro-vessels’ outgrowth, whereas ARV p17 expression induced a dramatic reduction of the sprouting response (Figure 3B).

These data strongly suggest that ARV p17 may act as an angiosuppressor by interfering with mechanisms underlying spontaneous angiogenesis and hampering EC responses to stimulation by FGF-2.

### 3.4. Recombinant GST-ARV p17 Inhibits Angiogenesis In Vitro, Ex Vivo and In Vivo

From a translational perspective, we decided to assess whether a recombinant ARV p17 protein may exert an angiosuppressive activity, as observed above for the transduced counterpart. To this aim, the recombinant ARV p17 protein was expressed in *E. coli* fused with a GST tag at the N-terminus (GST-ARV p17), purified to homogeneity, and the identity of this 43 kDa protein was confirmed by Western blot analysis using a goat anti-GST antibody (Supplementary Figure S1A, B).

ARV p17 is a nucleocytoplasmic shuttling protein [4]. Accordingly, GST-ARV p17 showed a cytoplasmic immunolocalization when HUVECs were incubated for 16 h with 10 ng/mL of GST-ARV p17 protein, and cells were immunostained using a polyclonal antibody to a synthetic peptide derived from the ARV p17 protein. No immunoreactivity was instead observed for cells incubated with recombinant GST, here used as a negative control (Supplementary Figure S1C).

Next, we performed experiments to assess whether the recombinant GST-ARV p17 protein, added exogenously to ECs cultures, induces the same angiosuppressive activity observed after nucleofection of the ARV p17 plasmid DNA-harboring expression vector. To this end, HUVECs were pretreated with GST-ARV p17 (10 ng/mL) for 24 h, whereas GST protein was used as a negative control.

**Figure 4:**
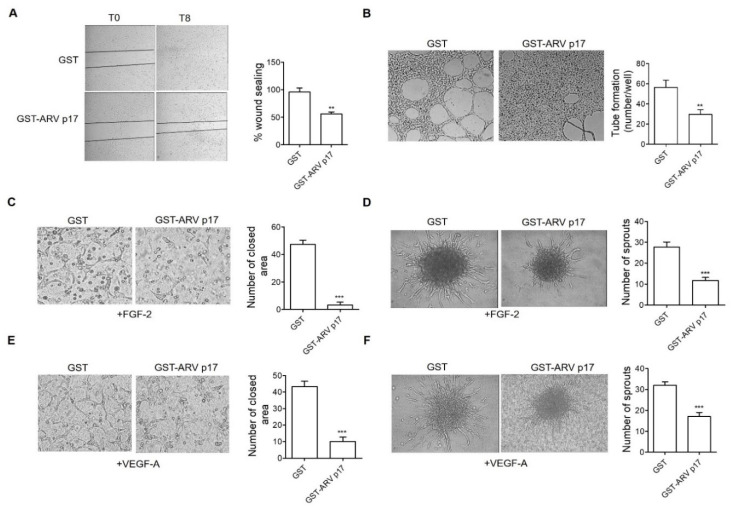
Effect of recombinant Glutathione S-Transferase (GST)-ARV p17 protein on angiogenesis. HUVECs were pretreated with 10 ng/mL of either recombinant GST or GST-ARV p17 for 24 h. (**A**) Wound healing assay to assess migratory activity of ECs. Confluent cell monolayers were scratched using a 200 μL pipette tip and cell migration was recorded by light microscopy 8 h after wound scratch (original magnification, 4×). The wound width was measured, and the relative wound area was calculated as the ratio of the remaining area at the 8 h time point to the 0 h starting point. Pictures are representative of one out of three independent experiments with similar results. Values are the mean ± SD of one representative experiment out of three with similar results, performed in triplicates. Statistical analysis was performed by Student’s *t* test. ** *p <* 0.01. (**B**) Matrigel assay to assess tube formation activity in GST- and GST-ARV p17-pretreated cells. Pictures were taken after 6 h of culture (original magnification, 4×). Closed rings were counted as a parameter for quantification of tube formation. Pictures are representative of one out of three independent experiments with similar results. Values are the mean ± SD of one representative experiment out of three with similar results, performed in triplicates. Statistical analysis was performed by Student’s *t* test. ** *p <* 0.01. (**C**) 3D Matrigel assay in the presence of FGF-2 (100 ng/mL) to assess the tube formation in GST- and GST-ARV p17-pretreated cells. Pictures were taken after 24 h from cell seeding (original magnification, 10×). The closed areas were counted as a parameter for quantification of tube formation. Pictures are representative of one out of three independent experiments with similar results. Values are the mean ± SD of one representative experiment out of three with similar results, performed in triplicates. Statistical analysis was performed by Student’s *t* test. *** *p <* 0.001. (**D**) Spheroid assay in the presence of FGF-2 (100 ng/mL) to assess sprout formation in GST- and GST-ARV p17-pretreated cells after 24 h (original magnification, 20×). The bar graph shows the average number/spheroid of EC sprouts. Pictures are representative of one out of three independent experiments with similar results. Values are the mean ± SD of one representative experiment out of three with similar results, performed in triplicates. Statistical analysis was performed by Student’s *t* test. *** *p <* 0.001. (**E**) 3D Matrigel assay in the presence of VEGF-A (100 ng/mL) to assess the tube formation in GST- and GST-ARV p17-pretreated cells. Pictures were taken after 24 h of culture (original magnification, 10×). The closed areas were counted as a parameter for quantification of tube formation. Pictures are representative of one out of three independent experiments with similar results. Values are the mean ± SD of one representative experiment out of three with similar results, performed in triplicates. Statistical analysis was performed by Student’s *t* test. *** *p <* 0.001. (**F**) Spheroid assay in the presence of VEGF-A (30 ng/mL) to assess sprout formation after 24 h in GST- and GST-ARV p17-pretreated cells (original magnification, 20×). The bar graph shows the average number/spheroid of EC sprouts. Pictures are representative of one out of three independent experiments with similar results. Values are the mean ± SD of one representative experiment out of three with similar results, performed in triplicates. Statistical analysis was performed by Student’s *t* test. *** *p <* 0.001.

As shown in Figure 4A,B, recombinant GST-ARV p17 inhibits the capacity of HUVECs to seal a mechanical wound of the cell monolayer and to form capillary-like structures when seeded on Matrigel-coated plates, whereas recombinant GST protein was ineffective, thus confirming the specificity of the inhibition.

To assess whether exogenously administered recombinant GST-ARV p17 protein was able to counteract the activity of well-known proangiogenic factors such as FGF-2 and Vascular Endothelial Growth Factor A (VEGF-A), we performed a 3D Matrigel assay in the presence of FGF-2 or VEGF-A (both at 100 ng/mL). As shown in Figure 4C,E, within 24 h of incubation, GST-pretreated cells formed a capillary-like network upon FGF-2 or VEGF-A stimulation. The activity of the two angiogenic factors was instead drastically reduced by pretreatment with the GST-ARV p17 protein. A similar angiosuppressive effect was exerted by GST-ARV p17 on HUVEC spheroids embedded in a type I collagen gel. Indeed, also in this case, the viral protein was able to inhibit EC sprouting induced by FGF-2 or VEGF-A (Figure 4D,F).

Taken together, these data demonstrate that ARV p17 induces a remarkable anti-migratory and antiangiogenic activity when expressed endogenously by transduced ECs as well as following its administration as an exogenous recombinant protein.

**Figure 5:**
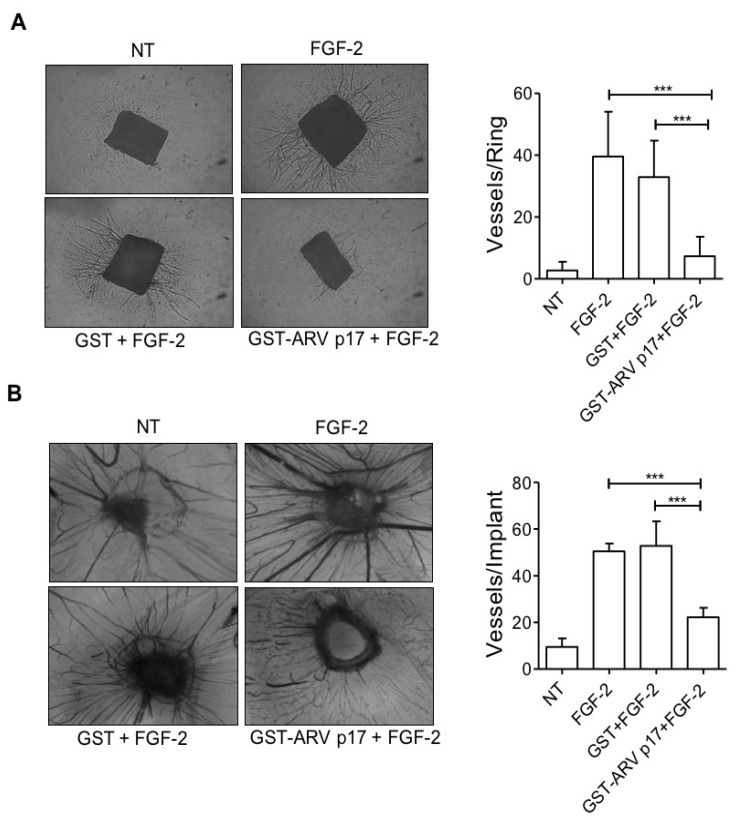
In vivo and ex vivo effects of ARV p17 on angiogenic process. (**A**) Mouse aortic rings, embedded in fibrin gel, were incubated for 24 h, as indicated. After 6 days, micro-vessels were observed, counted under a stereomicroscope and data were expressed as the number of sprouts per aortic ring (mean ± SD of 7–10 rings per experimental point). Pictures are representative of two independent experiments with similar results (original magnification, 4×). Statistical analysis was performed by one-way analysis of variance (ANOVA) and Bonferroni’s post-test was used to compare data. *** *p <* 0.001. (**B**) Micro-vessels converging towards the implant were counted at day 14 under a stereomicroscope. Pictures of CAMs are representative of two independent experiments with similar results (original magnification, 5×). Bars represent the mean ± SD of 4–5 eggs per experimental point in two independent experiments. Statistical analysis was performed by one-way ANOVA and Bonferroni’s post-test was used to compare data. *** *p <* 0.001.

Following these in vitro observations, the recombinant GST-ARV p17 protein was then tested for its capability to inhibit neovessel formation by ex vivo and in vivo experiments. To this aim, the effect of ARV p17 on angiogenesis was studied ex vivo using a murine aortic ring assay [25]. Aortic rings were treated with GST- or GST-ARV p17 for 24 h, and then cultured in the presence of FGF-2 (100 ng/mL) for 6 days. As expected, stimulation of aortic rings with FGF-2 strongly increased micro-vessel outgrowth that was significantly suppressed by pretreatment of the aorta rings with GST-ARV p17, but not by pretreatment with GST (Figure 5A). These data were confirmed in vivo using the chick chorioallantoic membrane (CAM) assay [26]. As shown in Figure 5B, a significant angiogenic response was promoted by 100 ng/embryo of FGF-2 in the form of numerous neovessels developing radially toward the implant in a “spoke-wheel” pattern (mean number of vessels/embryo: 50 ± 3) as compared to the untreated (NT) group (mean number of vessels/embryo: 9 ± 3). As expected, GST treatment did not affect FGF-2-induced vessel formation (mean number of vessels/embryo: 53 ± 9) that was instead significantly reduced (*p* < 0.01) by GST-ARV p17 treatment (mean number of vessels/embryo: 22 ± 3). These results further attest for the ability of ARV p17 to interfere with the mechanisms underlying induced angiogenesis, both ex vivo and in vivo.

### 3.5. ARV p17 Upregulates the Antiangiogenic Factor DPP4 in HUVECs

**Figure 6:**
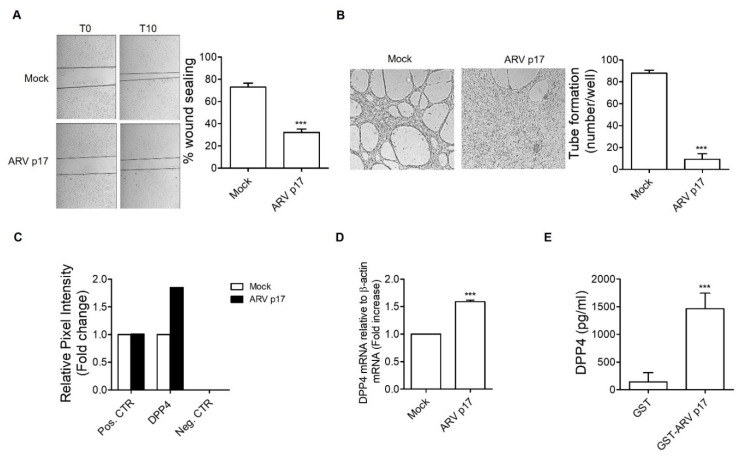
ARV p17-mediated release of Dipeptidyl Peptidase 4 (DPP4) expression. (**A**) Wound healing assay performed after HUVECs’ overnight stimulation with conditioned medium from Mock- or ARV p17-nucleofected HUVEC cells. Confluent cell monolayers were scratched using a 200 μL pipette tip and cell migration was recorded by light microscopy 10 h after wound scratch (original magnification, 4×). The wound width was measured, and the relative wound area was calculated as the ratio of the remaining area at the 10 h time point to the 0 h starting point. Pictures are representative of one out of two independent experiments with similar results. Values are the mean ± SD of one representative experiment out of two with similar results, performed in triplicates. Statistical analysis was performed by Student’s *t* test. *** *p <* 0.001. (**B**) Tube formation assay performed with HUVECs co-cultivated for 48 h with Mock- or ARV p17-nucleofected HUVEC cells. The pictures were taken 6 h after cell seeding (original magnification, 4×). Closed rings were counted as a parameter for quantification of tube formation. Pictures are representative of one out of two independent experiments with similar results. Values are the mean ± SD of one representative experiment out of two with similar results, performed in triplicates. Statistical analysis was performed by Student’s *t* test. *** *p <* 0.001. (**C**) Angiogenesis array performed with the supernatants of Mock- or ARV p17-nucleofected cells recovered 24 h post-nucleofection. Relative pixel intensity was calculated using ImageJ software and expressed as the mean of duplicate dots. Values are representative of one experiment out of two with similar results. (**D**) Analysis of DPP4 gene expression performed using quantitative real-time PCR in Mock- and ARV p17-nucleofected cells. Analysis of real-time PCR data were performed with the 2^-DDCt^ method using relative quantitation study software. Quantification of DPP4 mRNA was normalized according to the internal β-actin control. Values represent the mean ± SD of one representative experiment out of two with similar results, performed in triplicates. Statistical analysis was performed by Student’s *t* test. *** *p <* 0.001. (**E**) soluble (s) DPP4 levels in supernatants of HUVECs stimulated with GST-ARV p17 for 48 h were quantified with human sDPP4 ELISA. Supernatant from GST stimulated cells was used as a negative control. Bar graph displays the concentration of DPP4 determined by absorbance quantification. Values are the mean ± SD of one representative experiment out of three independent experiments with similar results, performed in triplicates. Statistical analysis was performed by Student’s *t* test. *** *p <* 0.001.

Our data demonstrates that ARV p17 interferes with FGF-2- and VEGF-A-induced neovascular responses, suggesting that this viral protein may promote a microenvironment unfavorable to neovessel formation. To test this hypothesis, we investigated the effect of the conditioned medium (CM) from ARV p17-transduced HUVECs on the angiogenic activity of naïve ECs. As shown in Figure 6A, pretreatment with the CM obtained from ARV p17-expressing ECs impaired the capacity of HUVECs to repair the wounded monolayer, whereas the CM from Mock HUVECs was ineffective. Moreover, when naïve HUVECs were co-cultured for 48 h on the collagen-coated upper insert well of a 0.4 μm pore-size transwell in the presence of ARV p17-expressing cells in the lower chamber, they lost the ability to form tube-like structures once detached and seeded on Matrigel. No effect was, instead, exerted by co-culturing naïve ECs with Mock HUVECs (Figure 6B).

To understand whether the antiangiogenic activity of ARV p17 was direct or mediated by other molecules, we analyzed the secretome of Mock and ARV p17-expressing HUVECs by using a human angiogenesis array. As shown in Figure 6C, the CM of ARV p17 HUVECs was characterized by higher levels of the antiangiogenic dipetidyl peptidase 4 (DPP4) when compared to the CM of Mock cells. No significant difference was instead detected for all the other antiangiogenic factors, like endostatin, vasohibin, pentraxin 3 and thrombospondin-1, probed by this array (data not shown). The increased levels of DPP4 protein secretion induced by ARV p17 transduction were paralleled by an increase in DPP4 mRNA expression, as assessed by Reverse Transcriptase-quantitative Polymerase Chain Reaction (RT-qPCR) analysis of ARV p17 versus Mock HUVECs (Figure 6D).

To confirm the capability of ARV p17 to promote DPP4 release by ECs, naïve HUVECs were incubated for 48 h in the presence of recombinant GST-ARV p17 or GST protein. As shown in Figure 6E, recombinant GST-ARV p17 induced a significant increase of soluble DPP4 (sDPP4) in the CM of treated HUVECs, as assessed by ELISA.

### 3.6. DPP4 Mediates the Antiangiogenic Activity of ARV p17

**Figure 7:**
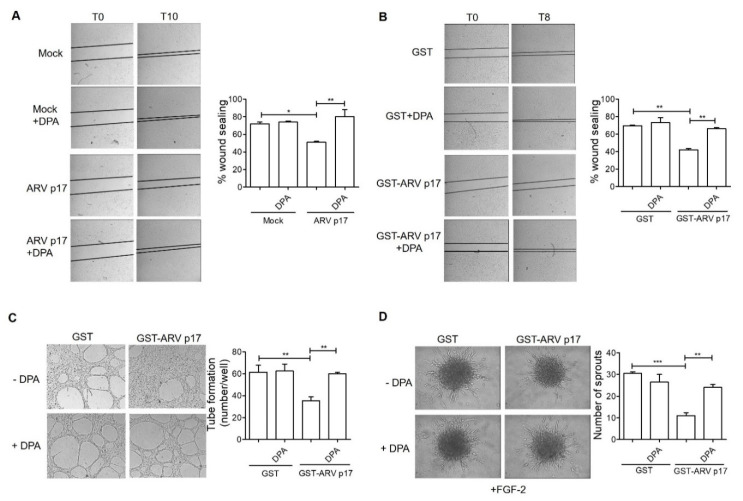
Effect of Diprotin A (DPA) on ARV p17 activities. (**A**) Wound healing assay in the presence of DPA (10 μM) to assess migratory activity of ECs pretreated with conditioned medium from Mock- and ARV p17-expressing cells (original magnification, 4×). The wound width was measured, and the relative wound area was calculated as the ratio of the remaining area at the 10 h time point to the 0 h starting point. Values are the mean ± SD of one representative experiment out of three with similar results, performed in triplicates. Statistical analysis was performed by one-way ANOVA and Bonferroni’s post-test was used to compare data. * *p <* 0.05; ** *p <* 0.01. (**B**) Wound healing assay in the presence of DPA (10 μM) to assess migratory activity of ECs pretreated with recombinant GST or GST-ARV p17 (10 ng/mL) (original magnification, 4×). The wound width was measured, and the relative wound area was calculated as the ratio of the remaining area at the 8 h time point to the 0 h starting point. Values are the mean ± SD of one representative experiment out of three with similar results, performed in triplicates. Statistical analysis was performed by one-way ANOVA and Bonferroni’s post-test was used to compare data. ** *p <* 0.01. (**C**) Tube formation assay in the presence of DPA (10 μM) to assess capillary-like structures formation in GST- and GST-ARV p17-pretreated cells. Pictures were taken after 6 h of culture (original magnification, 4×). Closed rings were counted as a parameter for quantification of tube formation. Values are the mean ± SD of one representative experiment out of three with similar results, performed in triplicates. Statistical analysis was performed by one-way ANOVA and Bonferroni’s post-test was used to compare data. ** *p <* 0.01. (**D**) 3D spheroid assay in the presence of DPA (10 μM) and FGF-2 (100 ng/mL) to assess sprout formation in GST- and GST-ARV p17-pretreated cells (original magnification, 20×). The bar graph shows the average number/spheroid of EC sprouts. Pictures are representative of one out of three independent experiments with similar results. Values are the mean ± SD of one representative experiment out of three with similar results, performed in triplicates. Statistical analysis was performed by one-way ANOVA and Bonferroni’s post-test was used to compare data. ** *p <* 0.01; *** *p <* 0.001.

To assess whether DPP4 upregulation plays a non-redundant role in mediating the angiosuppressive activity of ARV p17, HUVECs were pretreated with the CM from ARV p17-expressing ECs or with recombinant GST-ARV p17, whereas Mock and GST pretreated cells were used as negative controls, respectively. Then, cells were tested in different angiogenesis assays in the absence or the presence of an optimal concentration (10 μM) of the selective DPP4 inhibitor Diprotin A (DPA) [28]. As shown in Figure 7A,B, DPA fully rescued the inhibitory effect exerted by the CM from ARV p17 HUVECs or from recombinant ARV p17 protein on the migratory capacity of HUVECs in the mechanical wound healing assay, as well as their ability to form tubular-like structures when seeded on Matrigel (Figure 7C). In addition, DPA prevented the inhibitory effect exerted by ARV p17 on the capacity of HUVEC spheroids to form EC sprouts when embedded in a type I collagen gel in the presence of FGF-2 (Figure 7D).

**Figure 8:**
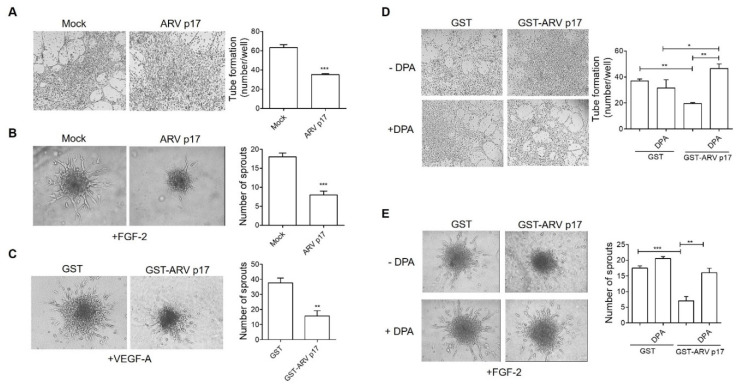
Effect of ARV p17 expression on Human Microvascular Endothelial Cells (HMVECs) angiogenesis. HMVEC cells were mock-nucleofected (Mock) or nucleofected with ARV p17-expressing plasmid (ARV p17). Angiogenesis was analyzed 48 h post-nucleofection (**A**,**B**). (**A**) HMVECs were seeded on BME-coated plates. Images were taken after 6 h of HUVEC culture on BME (original magnification, 4×). Closed rings were counted as a parameter for quantification of tube formation. Values are the mean ± SD of one representative experiment out of two with similar results, performed in triplicates. Statistical analysis was performed by Student’s *t* test. *** *p <* 0.001. (**B**) Sprouting of spheroids generated by Mock or ARV p17 HMVECs upon FGF-2 treatment. Pictures were taken after 24 h of culture (original magnification, 20×). The bar graph shows the average number/spheroid of EC sprouts. Values are the mean ± SD of one representative experiment out of two with similar results, performed in triplicates. Statistical analysis was performed by Student’s *t* test. *** *p <* 0.001. HMVECs were pretreated with 10 ng/mL of either recombinant GST or GST-ARV p17 for 24 h (**C**–**E**). (**C**) Spheroid assay in the presence of Vascular Endothelial Growth Factor A (VEGF-A) (30 ng/mL) to assess sprout formation after 24 h in GST- and GST-ARV p17-pretreated cells (original magnification, 10×). The bar graph shows the average number/spheroid of EC sprouts. Values are the mean ± SD of one representative experiment out of two with similar results, performed in triplicates. Statistical analysis was performed by Student’s *t* test. ** *p <* 0.01. (**D**) Tube formation assay in the presence of DPA (10 μM) to assess capillary-like structures in GST- and GST-ARV p17-pretreated cells. Pictures were taken after 8 h of culture (original magnification, 4×). Closed rings were counted as a parameter for quantification of tube formation. Values are the mean ± SD of one representative experiment out of two with similar results, performed in triplicates. Statistical analysis was performed by one-way ANOVA and Bonferroni’s post-test was used to compare data. * *p <* 0.05; ** *p <* 0.01. (**E**) Spheroid assay in the presence of DPA (10 μM) and FGF-2 (100 ng/mL) to assess sprout formation after 24 h in GST- and GST-ARV p17-pretreated cells (original magnification, 20×). The bar graph shows the average number/spheroid of EC sprouts. Pictures are representative of one out of two independent experiments with similar results. Values are the mean ± SD of one representative experiment out of two with similar results, performed in triplicates. Statistical analysis was performed by one-way ANOVA and Bonferroni’s post-test was used to compare data. ** *p <* 0.01; *** *p <* 0.001.

Since ARV p17 has been proven to exert anti-carcinogenic activity on human lung cancer cells, amongst others [10], we wondered whether the viral protein could also be active on human microvascular endothelial cells of lung origin (HMVECs) and whether also in this case, its effects were mediated by DPP4. To this aim, we performed key experiments by using HMVECs either transduced with ARV p17 or pretreated with GST-ARV p17. As shown in Figure 8A, ARV p17-expressing HMVECs lost their ability to form capillary-like structures when seeded on a Matrigel. Furthermore, ARV p17 expression inhibited the capacity of HMVEC spheroids to sprout in response to FGF-2 or VEGF-A (Figure 8B,C). Finally, as observed for HUVECs, the DPP4 inhibitor DPA almost completely rescued the inhibitory effect, exerted by recombinant GST-ARV p17 protein on HMVECs both in capillary-like structure formation assay (Figure 8D) and spheroid assay (Figure 8E).

Together, these findings indicate that ARV p17 is able to act as an angiosuppressive mediator on both macrovascular and microvascular human ECs and that its activity is mediated by the antiangiogenic enzyme DPP4.

## 4. Discussion

In the present study, we show the antiangiogenic property of the non-structural viral protein ARV p17, which occurs through the increased secretion of sDPP4. In particular, ARV p17, either endogenously expressed or exogenously administrated, is able to significantly inhibit EC migration, tube formation and sprouting in vitro, but also ex vivo and in vivo, as confirmed by aortic ring and CAM assays, respectively. Moreover, the viral protein is capable to suppress not only the physiological angiogenesis, but also the one induced by two different potent angiogenic agents, namely FGF-2 and VEGF-A, thus indicating that ARV p17 can act as an effective wide-spectrum antiangiogenic molecule.

Current antiangiogenic therapies use drugs which specifically target only a single proangiogenic molecule, even though tumors can activate alternative pathways to stimulate angiogenesis [29]. In particular, the most important and best-characterized proangiogenic molecular factors and signaling pathways involved in tumor neoangiogenesis are the members of the VEGF family, which are expressed at high levels in most tumors and involved in conditioning the microenvironment to impact on EC angiogenic activity [30]. Therefore, VEGF-mediated signaling has become one of the most promising antiangiogenic therapeutic targets in oncology for clinical treatment of human cancers and metastasis [31], especially when it is not difficult to find acquired or intrinsic therapy resistance associated with anti-VEGF monotherapeutic approaches. To date, a crucial need remains for the introduction of a next generation of antiangiogenic drugs which could simultaneously block different proangiogenic pathways by showing a wide clinical efficacy [32]. Within this context, ARV p17, by simultaneously interfering with both VEGF- and FGF-signaling cascades, could represent a new drug to be used alone or in combination with other drugs in producing an effective anti-cancer activity. It is worth noting that the mitogenic activity of FGF-2 is significantly more powerful than that of VEGF-A [33]. Since FGF-2 is involved in the resistance to VEGF-inhibition [34,35,36], it represents a novel target for antiangiogenic drugs possibly also endowed, as for ARV p17, with a simultaneous anti-VEGF activity.

Numerous proteins and chemical molecules have been found to inhibit angiogenesis, but the underlying mechanism of their action is poorly understood and their outcome in clinical trials remains unpredictable or sometimes, disappointing [37]. In this study, we highlighted the molecular mechanism by which the viral protein ARV p17 exerts its antiangiogenic activity. In particular, we identified DPP4/CD26, a serine exopeptidase expressed on different HMVECs (i.e., in liver, kidney, lungs and brain) [38], as the molecule responsible for the ARV p17 antiangiogenic function. DPP4 is mainly anchored onto the cell membrane and then released in an active soluble form (sDPP4) in the culture supernatant and in different biological fluids [39,40,41]. In our study, we demonstrated that ARV p17 increases sDPP4 in the ECs supernatants by a still uncovered mechanism. Matrix metalloproteases and kallikrein-related peptidase 5 have been implicated in its shedding [42,43]. Studies are warranted in the future to understand the role of these molecules in the ARV p17-induced sDPP4 release.

DPP4 is a multifunctional protein, which possesses the capability, through its intrinsic peptidase activity, to inactivate or degrade many substrates, including chemokines involved in cell migration and tumor metastases [44,45]. Currently, it is not easy to exactly determine the role of DPP4 in carcinogenesis, since it can act as a tumor promoter or suppressor [38,46] and its role is essentially dependent on tumor type and localization, cell type and microenvironment. However, DPP4 displays an antioncogenic function in those tumors linked to the CXCL12/CXCR4 axis, which is known to support neovascularization, tumor growth and metastasis during cancer development [28,47,48,49,50]. In this context, different studies suggested that CXCL12 degradation by DPP4 is involved in regulation of the metastatic process [51,52] and that the removal of two N-terminal amino acids from CXCL12 reduces the chemokine affinity for the CXCR4 receptor and, consequently, its activity [53,54]. Moreover, DPP4 is known to suppress angiogenesis by directly interacting with CXCR4 and downregulating its mRNA and protein level [48]. Different studies highlighted that inhibition of tumor progression also occurred in the absence of DPP4 enzymatic activity [48,55], as an increased DPP4 gene expression being sufficient enough to revert the malignant tumor phenotype [28,48,56,57]. Therefore, we can hypothesize that the increased DPP4 mRNA expression triggered by ARV p17 (Figure 8D) may also play a role in modulating the CXCL12/CXCR4 axis to impair angiogenesis. Moreover, our results showing the capability of ARV p17 to block FGF-2-induced angiogenesis through the involvement of DPP4 are in line with the previous studies showing the ability of DPP4 to inhibit the malignant phenotype of prostate cancer cells by blocking FGF-2 signaling [58].

To the best of our knowledge, the ability of ARV p17 to condition the microenvironment and impact on angiogenesis through an increased expression of sDPP4 is completely new and suggests a peculiar activity of the viral protein. ARV p17 is known to inhibit cell growth of different cancer cell lines both in vitro and in vivo by triggering cell cycle retardation [5,10,12]. Thus, the “two compartments” activity of ARV p17 may provide anticancer therapeutic benefits not only in terms of oncosuppressive effects on tumor cells but also by inhibiting the neovascularization process, hence hampering the tumor parenchymal/stromal crosstalk.

In conclusion, the complex antitumor activities of ARV p17 may open the way to a new promising field of research aimed to develop new therapeutic approaches for treating tumor and cancer metastasis.

## 5. Conclusions

In this study, given the involvement of ARV p17 in cell growth and tumorigenesis, we explored its capability to regulate angiogenesis, which is one of the fundamental processes in tumor growth. Our data highlights that ARV p17, either endogenously expressed or exogenously administrated, can act as an effective antiangiogenic molecule on ECs by suppressing the angiogenesis also mediated by two potent inducers, such as VEGF-A and FGF-2. Moreover, we demonstrate that ARV p17 exerts its antiangiogenic function through DPP4 action.

Overall, our findings define this viral protein as a promising drug to develop new therapeutic approaches for cancer therapy.

## Supplementary Materials

The following are available online at https://www.mdpi.com/2073-4409/10/2/259/s1, Figure S1A: SDS-PAGE analysis of purified recombinant GST-ARV p17 protein, Figure S1B: Western blot analysis of recombinant GST and GST-ARV p17, Figure S1C: Immunofluorescence analysis of recombinant GST-ARVp17 in HUVEC cells.
