# Supplementary material for: Avian Reovirus P17 Suppresses Angiogenesis by Promoting DPP4 Secretion
Source: Cells. 2021 Jan 28;10(2):259. doi: 10.3390/cells10020259 (PMC7911508; doi:10.3390/cells10020259)
Supplement: Supplementary file 1 [file cells-10-00259-s001.pdf]

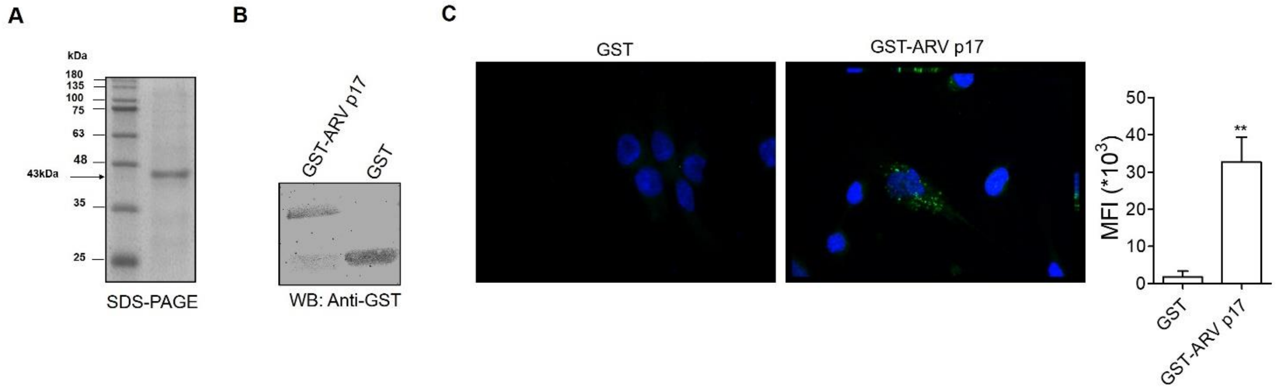


**Figure S1.** Recombinant GST-ARV p17 characterization **(A)** Purified recombinant GST-ARV p17 protein analyzed by SDS-PAGE electrophoresis and stained with Coomassie. (**B)** Western blot analysis of recombinant GST and GST-ARV p17 by goat anti-GST antibody. **(C)** HUVECs were treated for 16 h at 37°C with 10 ng/ml of recombinant GST or GST-ARV p17. Images display GST-ARV p17 signals in green and cell nuclei in blue. Bar graph displays mean fluorescence intensity observed for ARV p17 signal quantification. Pictures are representative of five selected fields/sample (original magnification, 63×). MFI = Mean Fluorescence Intensity. Statistical analysis was performed by Student's t test. ** $P < 0.01$.
